# Policy brief Belgian EBCP Mirror Group patient and citizen engagement

**DOI:** 10.1186/s13690-024-01370-w

**Published:** 2024-08-26

**Authors:** Wannes Van Hoof, Gabrielle Schittecatte, the Thematic Working Group on Patient & Citizen Engagement

**Affiliations:** Cancer Centre, Sciensano, Brussels, Belgium

**Keywords:** Policy recommendations, Patient involvement, Ethics, Data

## Abstract

Rights, preferences, needs and expectations of patients and citizens can only be respected and addressed if they are well understood. As such, a continuous, systematic and formalised dialogue between patients, citizens and policy makers is required to ensure ethical and socially appropriate cancer prevention, diagnostics, treatment and care. Relying on donations and project-based funding is not a sustainable way to ensure patient involvement and representativeness in policy. Patient organizations need long term, structural support to fulfil their role as patient representatives and support network in order to deliver the best possible service and to play their role as a professional representative of their disease-specific community. Inequalities can only be tackled if they are properly identified. This requires the definition of appropriate determinants fit for (inter)national comparison and extension and linkage of good quality data registries for cancer that allow the monitoring these inequalities.

## Background

### Introduction

The objective of this policy brief is to outline the major activities and initiatives related to patient and citizen engagement in cancer care, analyse and detail the remaining gaps and their consequences, then conclude with key recommendations for policy and decision-makers to improve the status quo. This brief is not a literature review or a new study, but it represents the perspective of different actors on the optimal use of patient and citizen engagement in Belgium based on the experience of several ongoing European projects.

### Methodology

This brief, including the key activities, persistent gaps, and recommendations is the result of a participative process, including group meetings and workshops with the *Citizen and Patient Engagement Belgian-EBCP Mirror Group* and patient organizations. During these meetings and workshops, perspectives were gathered on the current state of play, added value of ongoing Belgian and European projects, and, building on the former two points, persistent gaps in patient and citizen engagement. These discussions were analysed to develop the following policy brief.

The working group currently consists of more than 50 members, with representatives from patient organizations, NGOs, hospitals, industry, professional organizations, research institutes, registries and universities. A full list of members can be found on the corresponding tab of the stakeholder mapping accompanying this document.

## Main text

### Issue overview

In many national and international funding programs, including the EBCP and the EU4Health programme, patient and citizen engagement is considered as an important part of new projects. This is a step in the right direction, but it needs to be complemented by a sustainable strategy to involve patients and citizens structurally and continuously. This is particularly true as cancer concerns the entire population; all individuals are susceptible to cancer, and the entire population is affected by screening, vaccination and prevention programs. Moreover, cancer care is a societal challenge that touches upon many sensitive topics such as reimbursement of expensive drugs, the use of genomic information, behaviour change, surveillance, data protection, merging therapeutics and research and even the very definition of what it means to live a good or healthy life. Such challenging topics require the engagement of patients and citizens to determine what our values are.

Disease-specific patient organisations support patients and their families by better understanding their disease and by helping them navigate and use the healthcare system. In this way, patients are more engaged in their treatment process, receive better support and have both better health outcomes and higher satisfaction of their treatment.

Patient and citizen engagement is essential to prevent ‘shadowboxing’ between health professionals, policy makers and targeted populations. When concepts like privacy, consent, control, health, risk, care, survivorship, dignity, etc. mean something different to each of these parties, it is possible for everyone to fully intend to respect each other’s rights and preferences and do the exact opposite. Each stakeholder has by definition a different perspective and will have a different view on priorities, gaps and best practices. Without a continuous dialogue, perspectives may not align; this way we would revert back to twentieth century strategies, like unidirectional awareness-raising campaigns, persuasion and top-down behaviour change campaigns that do not work for a twenty-first century public.

### Projects, gaps & policy recommendations

#### GAP: citizen engagement in health data management & use

The *Healthy Data (TEHDAS*) project aims to discuss with citizens about the ethical, legal and societal implications of the creation of the European Health Data Space (EHDS) and how they want to be involved in its governance. The Health Data Agency is a new federal health data governance institute that aims to streamline the primary and secondary use of health data in Belgium and the implementation of the EHDS. This project identified what citizens themselves believe to be the main ethical challenges of secondary use of health data and what their role should be in data governance within the EHDS framework.

With the TEHDAS project, an online communication campaign covering social media, newsletters, blogs, mailing lists, etc. supported by partnering patient organizations and civil society groups reached nearly 2 million individuals were put in contact with informative materials about the secondary use of health data. All informational materials invited citizens to share their opinion on a free form online deliberative platform. 6000 citizens contributions were gathered, mainly from Belgium, the UK and France where the leading institutions were based. This showcases that dialogue between citizens and decision-makers on contentious subjects to improve policy is possible with engagement efforts. A qualitative analysis of these citizens voices was used to construct 12 specific policy recommendations for the EU [1].

However, even though the current EHDS proposal explicitly refers to the need for stakeholder and patient engagement, organized by the national health data authorities and health data access bodies, no guidance has been given for how this should be accomplished.

#### Policy recommendation—improve citizen participation in health data

A continuous dialogue between patients, citizens and policy makers is required to ensure ethical and socially appropriate cancer prevention, diagnostics and care. Rights and preferences can only be respected if they are well understood, preferably by cancer type. Bottom-up approaches to understand rights and preferences also help avoid technology driven conversations, in which rights and preferences of citizens may be lost.

In order to achieve this, we propose building a governance framework based on citizen’s values. For example, in France, citizens are actively involved every 7 years in a fundamental review of the law on Bioethics and policy makers need to respond to their input in open parliamentary sessions [2]. This governance framework is necessary as citizens are both the source of the data and the ultimate users (individual and societal benefit) of the EHDS.

#### Policy recommendation—address inequalities through improved data use

Currently, existing health data are not used effectively. When looking by type of cancer, inequalities, difference in outcomes and best practices within Belgium become immediately clear and can be remediated. National and regional registries need to be able to collect, store and link data on cancer prevention, diagnosis, treatment, care organization, as well as survivorship to identify inequalities and their determinants. Decisions on when and how to address inequalities are more challenging for national registries that face limitations in data collection, linkage and the creation of data dashboards by type of cancer, … If such registries and data collection are defined at the European level (e.g. the European Cancer Inequalities Registry), then it is important that these can be tailored to be fit for purpose at national and regional levels. Such data and dashboards would also facilitate patient organisations in addressing these inequalities through their work, alongside other health system actors.

#### GAP: patient & citizen involvement by design

Within the Joint Action on Networks of Expertise (JANE), the goal of the transversal task force on patient involvement is to design strategies and methods to involve patients by design in the new European networks of expertise on cancer. The methods designed in the JANE project could produce case studies that improve our understanding of how to strengthen this. Additionally, the new networks of expertise in cancer facilitated by JANE will be designed with structural patient engagement in mind. This will empower patients and inspire new collaborations, which can make care more relevant, adapted, and sustainable, while also improving overall quality of care in the health system.

Currently in Belgium, there are no formal structures that facilitate the engagement of patients in discussions held by networks of expertise. The willingness to increase participation has been stated frequently, but no framework has been established to indicate who should participate, when, at what level, and for what purpose.

Examples of patient involvement in design of projects is present elsewhere. For example, at the EU level patient organisations participated in the design of the care pathway for colorectal and pancreatic cancer (iPAAC). With the current collaboration models, patient organizations play a role and are co-designing cancer care and research, with hospitals [3]. Such collaboration and coordination across patient organizations and NGO’s about cancer exists infrequently in Belgium.

Moreover, patient organizations operate largely independently from healthcare professional networks in oncology. They are rarely involved in the design of cancer policies or research, but often consulted after the fact. The consequences of delayed and diminished participation may be diminished trust, increases in health inequalities, and care offered that is not adapted to needs, with decreased quality of care experienced by patients [4, 5]. This includes important non-clinical aspects like nutrition, physical activity or access to social services.

The one ‘patient organization’ does not exists. There are many types that all strive to achieve different purposes. To achieve good governance in this domain does not imply a strive towards unification or harmonization, but a strategy that allows the harvesting of the wealth of experiences and approaches and the facilitation of collaboration.

#### Case study—building on citizen engagement initiatives for cancer genomics

The *CAN.HEAL* project combined results from various citizen engagement initiatives with the legal framework designed within the B1MG project to create a trustworthy data environment for secondary use of health data in a cancer genomics data space. Results from iPAAC, the ‘My DNA, everybody’s business’ citizen forum, and the DNA debate (all organized by Sciensano) give a clear indication regarding the Belgian population’s attitude towards the use of genomic information in healthcare.

Using the above engagement approaches, citizens’ values will be respected in policies on genomics use, and in turn this will help increase public trust, improve understanding of genomics in cancer (benefits and risks) and support for the investment in and implementation of genomic technologies in oncology. As consequence, this may contribute to the more successful roll-out and scaling-up of the use of such technologies to improve efficacy of care in Belgium. At the European level, CAN.HEAL will help identify ethical, legal and societal challenges that other stakeholders do not, but that are relevant to determine the acceptability of certain practices and technologies.

Despite these advances on engaging with citizens on sensitive topics such as genomics, results from citizen engagement initiatives, even when they produce clear policy outputs, are rarely really taken into account when new policies are designed [6, 7].

#### Policy recommendation—improve involvement of patient organizations in care and care organisation

Most patient organizations need further financial and technical support to contribute to bridging the gap between scientists, health professionals, policy makers and the patient, and even the general public. There are not enough incentives for patient organizations to participate in steering groups, patient committees, think tanks or other projects with high relevance for governance. Without their participation relevance of policies, and their smooth uptake will decrease. The way of involving patient organisations should be co-constructed with patient representatives or patient experts, to develop a culture and framework where participative methods are implemented by design.

## Data Availability

Not applicable.

## Abbreviations

CAN.HEAL: Building the EU cancer and public health genomics platform
EBCP: Europe’s Beating Cancer Plan
EHDS: European Health Data Space
iPAAC: Innovative Partnership for Actions Against Cancer
JANE: Joint Action on Networks of Expertise in Cancer
NGO: Non-governmental organization
TEHDAS: Towards the European Health Data Space (Joint Action)
